# Social inequalities in malaria knowledge, prevention and prevalence among children under 5 years old and women aged 15–49 in Madagascar

**DOI:** 10.1186/s12936-015-1010-y

**Published:** 2015-12-12

**Authors:** Sean A. P. Clouston, Josh Yukich, Phil Anglewicz

**Affiliations:** Program in Public Health and Department of Preventive, Population and Family Medicine, Stony Brook University, 101 Nichols Rd., Health Sciences Center #3-071, Stony Brook, NY 11794 USA; Department of Tropical Medicine, Tulane University School of Public Health and Tropical Medicine, 1440 Canal St., Ste. 2301, New Orleans, LA 70112 USA; Department of Global Community Health and Behavioral Sciences, Tulane University School of Public Health and Tropical Medicine, 1440 Canal St., Ste. 2210, New Orleans, LA 70112 USA

**Keywords:** Malaria, Socio-economic factors, Social epidemiology, Epidemiology, Global health

## Abstract

**Background:**

Approximately 15 % of all deaths in Africa among children under five years old are due to malaria, a preventable and treatable disease. A prevailing sociological theory holds that resources (including knowledge, money, power, prestige, or beneficial social connections) are particularly relevant when diseases are susceptible to effective prevention. This study examines the role of socioeconomic inequalities by broadly predicting malaria knowledge and use of preventive technology among women aged 15–49, and malaria among children aged 6–59 months in Madagascar.

**Methods:**

Data came from women aged 15–49 years (N = 8279) interviewed by Madagascar’s 2011/2013 Malaria Indicator Studies, and their children aged under five years (N = 7644). Because geographic location may be associated with socioeconomic factors and exposure to malaria, multilevel models were used to account for unobserved geographic and administrative variation. Models also account for observed social, economic, demographic, and seasonal factors.

**Results:**

Prevalence among children four years old and younger was 7.8 %. Results showed that both mother’s education and household wealth strongly influence knowledge about and efforts to prevent and treat malaria. Analyses also revealed that the prevalence of malaria among children aged 6–59 months was determined by household wealth (richest vs poorest: OR = 0.25, 95 % CI [0.10, 0.64]) and maternal education (secondary vs none: OR = 0.51, 95 % CI [0.28, 0.95]).

**Conclusions:**

Malaria may be subject to socio-economic forces arising from a broad set of behavioural and geographic determinants, even after adjusting for geographic risk factors and seasonality. Nearly 21 % of the sample lacked primary schooling. To improve malaria reduction efforts, broad-based interventions may need to attack inequalities to ensure that knowledge, prevention and treatment are improved among those who are most vulnerable.

**Electronic supplementary material:**

The online version of this article (doi:10.1186/s12936-015-1010-y) contains supplementary material, which is available to authorized users.

## Background

Across Africa, 15 % of all deaths among children under five years old (2010 est.) was due to malaria [1]. Since 2000, over half of malaria-endemic countries have succeeded in reducing malaria incidence by 75 %. The worldwide risk of malaria has decreased by 45 % for all ages, and 51 % among children under 5 years old [2]. Such reductions may be broadly beneficial, as attempts to reduce malaria, such as improved healthcare or better bed net usage, may also be effective at malarial sequelae such as pre-term birth, anaemia, as well as long-term impairments of motor and cognitive functioning [3].

Disease control is not without consequence. Fundamental cause theory holds that individuals and families use resources (e.g., knowledge, money, power, prestige, and beneficial social connections) in situ to influence survival [4, 5]. With respect to malaria, social inequalities are likely to arise through a number of mechanisms including the cost of treatment [6], diffusion of information about malaria [7], uptake of preventive technology, such as bed nets [8], exposure and treatment for malarial symptoms [9], susceptibility [10], and because malaria may in turn burden communities and increase poverty. Insofar as preventive technologies are important, inequalities across these mechanisms could ultimately result in inequalities in malaria prevalence and mortality [11].

Globally, the literature consistently suggests that poverty and malaria are associated both at the national and local levels [12, 13], and even suggest that development may help to reduce malaria [14]. However, evidence linking socioeconomic inequalities to malaria has been mixed [15]. For example, a study in Southeast Nigeria found that malaria was more common in richer households, though also noting that malaria was often self-diagnosed and those with more socio-economic resources had fewer difficulties accessing health care than those with fewer resources [16]. A study of children in Ghana, Nigeria, Kenya, and Sierra Leone showed that children in the wealthiest households were least likely to report fevers [17]. Such mixed results might occur when multiple layers of inequality compound to determine risk [18, 19]. For example, while knowledge about disease prevention may be subject to social inequalities, they may also be related to geographic inequalities that determine local risk of health outcomes and efforts to improve public health [20]. Moreover, those who are poor tend to live in more rural areas that are further away from healthcare facilities [21, 22].

## Methods

### Setting

Madagascar is an island nation with 23 million inhabitants off the Eastern coast of Africa, where 7 % of the child mortality rate (58/1000 live births in 2012) was attributable to malaria [23]. While poverty in Madagascar is high, 81.3 % of the population lives on less than $1.25 a day, socioeconomic inequalities are relatively common. The World Bank [24] reports that the Gini coefficient in Madagascar is 44.1, which is nearer than Sweden (25—egalitarian) or South Africa (70—unequal) to the global average. Approximately 20–25 % of women aged 15–49 lack primary schooling [20]. Prevalence of malaria remains high in Madagascar at 168.8/10,000.

Within Madagascar, the role of social inequalities remains unclear: one study finds that wealth and education determined knowledge about malaria, but not bed net ownership [25], while another found that proximity to healthcare facilities explained inequalities in childhood mortality [22]. In part, inequalities arise because healthcare, though being free for the poor, is far away (40 % of the population lives >5 km from a health centre), charges for incidentals including bed sheets, is underfunded ($11/capita spent on health), and lacks sufficient numbers of healthcare workers [26]. Contemporary reports further suggest the situation is getting increasingly dire for the poor as funding is cut further and healthcare workers choose to work in cities rather than in rural areas [27]. As a result, individuals with higher socio-economic status in Madagascar are more likely to be tested for malaria, and to receive artemisinin-combination therapy (ACT), though prior analyses suggest that ACT will only be used to treat malaria in 3 % of all Malagasy children with fevers [28].

In this paper, nationally representative data were used to examine the extent to which social inequalities are associated with knowledge about, treatments for, and the prevalence of malaria in Madagascar. Further, because malaria is heterogeneously distributed geographically, multilevel modelling was used to address that clustering of risk in order to examine the association between the distribution of socio-economic resources and multiple mechanisms of malaria prevention and control among children under 4 years old, who are most vulnerable to malaria.

### Data

Data from Madagascar’s Malaria Indicators Study in 2011 and 2013 were pooled for analysis [29, 30]. The MIS is a multistage stratified sample of 594 (264 in 2011 and 284 in 2013) communities randomly distributed throughout 109 administrative districts throughout Madagascar. The full MIS includes 79,357 residents of Madagascar aged 0–98. Analysis of knowledge and preventive efforts was examined among all 8311 female respondents aged 15–49, whose knowledge and efforts are most likely to influence risk in children. Full data were available for 8279. Of these women, 68.4 % had children under five years old living in the home, 21.4 % had living children aged five and older, 7.2 % had never given birth, 2.1 % had no children but were currently pregnant, and 0.9 % had given birth but had no living children. For analyses of prevalence, blood testing was used to examine malaria prevalence was carried out among children under 4 years old (N = 7644), who were most vulnerable to malaria, and prevalence information was then linked to the mother’s observed indicators.

### Malaria indicators

To indicate malaria knowledge, women were identified if they were aware of four aspects of malaria epidemiology, that (1) mosquitoes transmit malaria, (2) fever is the primary symptom for malaria, (3) children are particularly vulnerable to malaria, and (4) bed nets prevent malaria. To examine malarial prevention, mothers were identified if they reported that each child under five years old had used a bed net the previous night. Mothers were further identified if they reported receiving at least two doses of intermittent preventive treatment (IPT) during pregnancy for each child who was currently aged two years old or younger. Children were identified who had been brought in for treatment during a child’s most recent fever. Finally, prevalence of malaria in children aged 6–59 months was measured microscopically: thick blood spots were delivered to the Malaria Research Unit at Madagascar’s Institut Pasteur where blood smears were microscopically examined for the presence of malaria for all children aged 6–59 months whose parents consented (96 % of parents) to testing.

### Socio-economic status

Mothers’ education was measured in three categories: no schooling, primary schooling or secondary schooling. Household wealth was measured using quintiles from the standard index of household wealth, which used principal components analysis of indicators of ownership of consumer items such as fans or televisions and characteristics of the dwelling such as type of flooring or source of drinking water [31]. For mapping purposes, the percentage of mothers without a primary school education who were living in households with wealth below the 40th percentile was used.

### Covariates

External phenomena may independently influence knowledge about malaria and may also be associated with socio-economic inequality. Models were therefore adjusted for factors likely influencing socio-economic status and malaria-related outcomes, including mother’s age, whether respondents were currently pregnant, whether households were in rural areas, year and month of interview, and child’s age and sex. Seemingly unrelated regression was used to test differences in log-odds ratios in order to examine model fit.

### Analysis

In Madagascar, child mortality is influenced by geographic factors affecting the spread of disease, as well as administrative factors such as number of and spread of healthcare centres [32]. Analyses provided descriptive characteristics: poverty and malaria prevalence rates were mapped to provide regional context; Pearson’s correlation coefficients were estimated to compare these aggregate distributions at the district level. Modelling was done in a two-step manner. First, analyses were done using weighted logistic regression. Logistic regression is a strategy that reliably accounts for observed factors (sometimes called ‘fixed effects’), such as socio-economic status and education. Weighting, in this instance, accounts for the complex cluster-sampled design.

Multilevel logistic models (MLLM) were then used to examine the influence of socio-economic indicators on risk factors, while implicitly accounting ‘random intercepts’, which account for shared contextual, but largely unobserved, heterogeneity at theoretically driven levels of analysis. As such, the following statistical model was fit using restricted maximum likelihood estimation:

$$\ln \left( {\frac{{\pi_{dch} }}{{1 - \pi_{dch} }}} \right) = \beta_{0} + \beta_{1} SES + \beta_{2} E + \beta_{k} X_{k} + \gamma_{d00} + \gamma_{0c0} + \gamma_{00h} + \varepsilon_{dch}$$

In the above equation, *β*_0_ references the population average prevalence odds, fixed effects estimators include: socio-economic status (SES), education (E) and a number (*k*) of observed covariates (*X*_*k*_*)*. Analyses incorporated information at two or three possible nested contextual levels of analysis (noted by sub-script d, c, and h), which account for different types of unobserved variability that could be associated with both socio-economic status and malaria risk. Analyses among women account for variability at district (d) and community (c) levels, while analyses among children additionally accounted for variability at household (h) levels. At district level, random intercepts (*γ*_*d00*_) were used to account for administrative differences, including public health efforts and availability of healthcare resources. At community-level, random intercepts (*γ*_*0c0*_) accounted for variation in risk of malaria, incorporating altitude, weather, proximity to the coast, distance to the nearest healthcare centre, and quality of nearby healthcare facilities and also for the cluster-sampled design. At the household level, random intercepts (*γ*_*00h*_) account for shared influences, such as parents’ unobserved beliefs or personality traits, which may influence childhood outcomes.

MLLM provides estimates that are unbiased to data that are “missing at random,” defined broadly as being captured explicitly by fixed effects or implicitly by random effects estimators. Pseudo-R^2^ was provided to examine model fit. Analyses were implemented in Stata 13.1/IC.

### Sensitivity analyses

To examine whether MLLM improved on model fit, fit of MLLM was compared to logistic regression. To test the sensitivity of analyses to this possibility, a Heckman correction was used to estimate the lack of data due to the death of a child [33]. Because sex and age of household head often proxy social standing of a household, models examine the benefit of incorporating sex and age of household health as well as household size. Finally, the sensitivity of results to the type of test by analysing malaria cases identified using rapid diagnostic testing: specifically, CareStart*™* (AccessBio) rapid diagnostic tests (RDTs) were used to indicate whether children had malarial antigens (HRP2 or pLDH) present in capillary blood; when RDTs showed positive results, children were immediately provided with free ACT by MIS staff.

## Ethics

These data represent secondary data analyses of publicly available de-identified data. As such, these analyses are not human subjects research.

## Results

Table 1 provides characteristics of the overall sample. Demographic indicators show that more than one-fifth of women reported no formal education, and most lived in rural areas. Examining malaria knowledge, sample characteristics revealed substantial variation in mothers’ knowledge of and use of disease prevention. For example, almost a third did not know that mosquitoes transmit malaria, while just over half recognized that children were vulnerable and that bed nets could be used to prevent malaria. Moreover, fewer than half had used an antimalarial during pregnancy or reported seeking treatment for a child’s last fever. On average, women were interviewed in April.

**Table 1:** Descriptive characteristics, Madagascar Malaria Indicator Survey 2011, 2013

Unit of analysis	Variable	%	
Woman	Mosquitoes transmit malaria	72.24	
Woman	Children are vulnerable	51.01	
Woman	Fever is a primary symptom of malaria	71.70	
Woman	Bed nets prevent malaria	59.19	
Mother	IPTp used during last pregnancy	46.14	
Child aged 0–4	Tests positive for malaria	7.78	
Child aged 0–4	Child slept under a treated net prior night	69.35	
Child aged 0–4	Child taken for treatment during last fever	49.68	
Woman	Household wealth		
	Poorest	18.68	
	Poorer	20.58	
	Middle	19.45	
	Wealthier	19.80	
	Wealthiest	21.49	
Woman	Educational attainment		
	None	21.80	
	Primary	49.72	
	Secondary and higher	28.49	
Woman	Rural household	89.90	
Woman	Currently pregnant	7.62	
Woman	Male child	50.79	
		Mean	SD
Woman	Age in years	30.97	8.89
	Month of interview	4.75	0.83
Child aged 0–4	Child’s age in months	29.63	17.51

Sample sizes vary depending on the sample being analysed. IPTp used during pregnancy is observed only among children aged 0–4 in the home. Child taken for last fever is only observed among children aged 0–4 who had had fevers in the past year

**Fig. 1:**
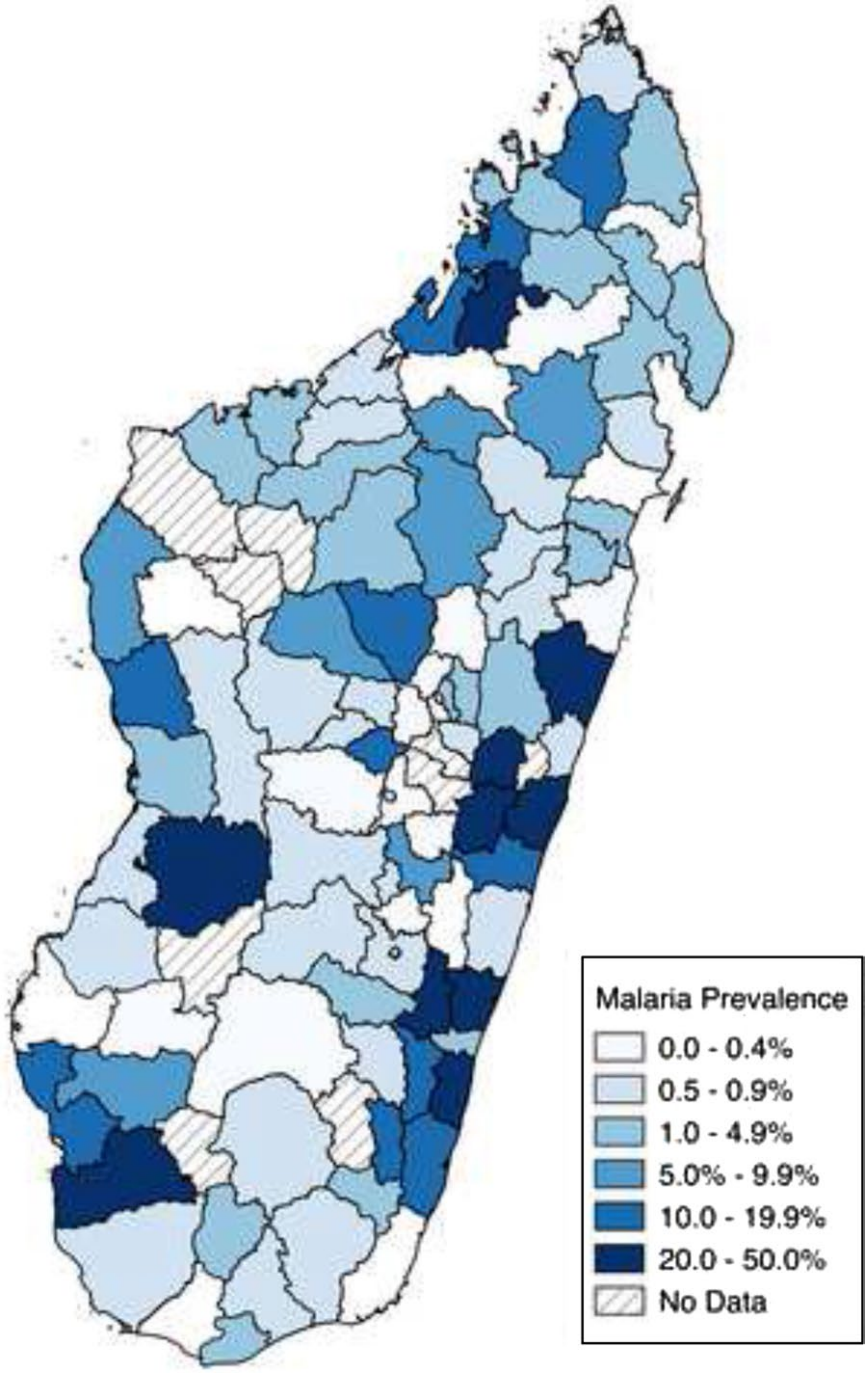
Malaria prevalence (% of children aged 6–59 months with malaria) at the district level. Malaria Indicators Survey 2011, 2013

**Fig. 2:**
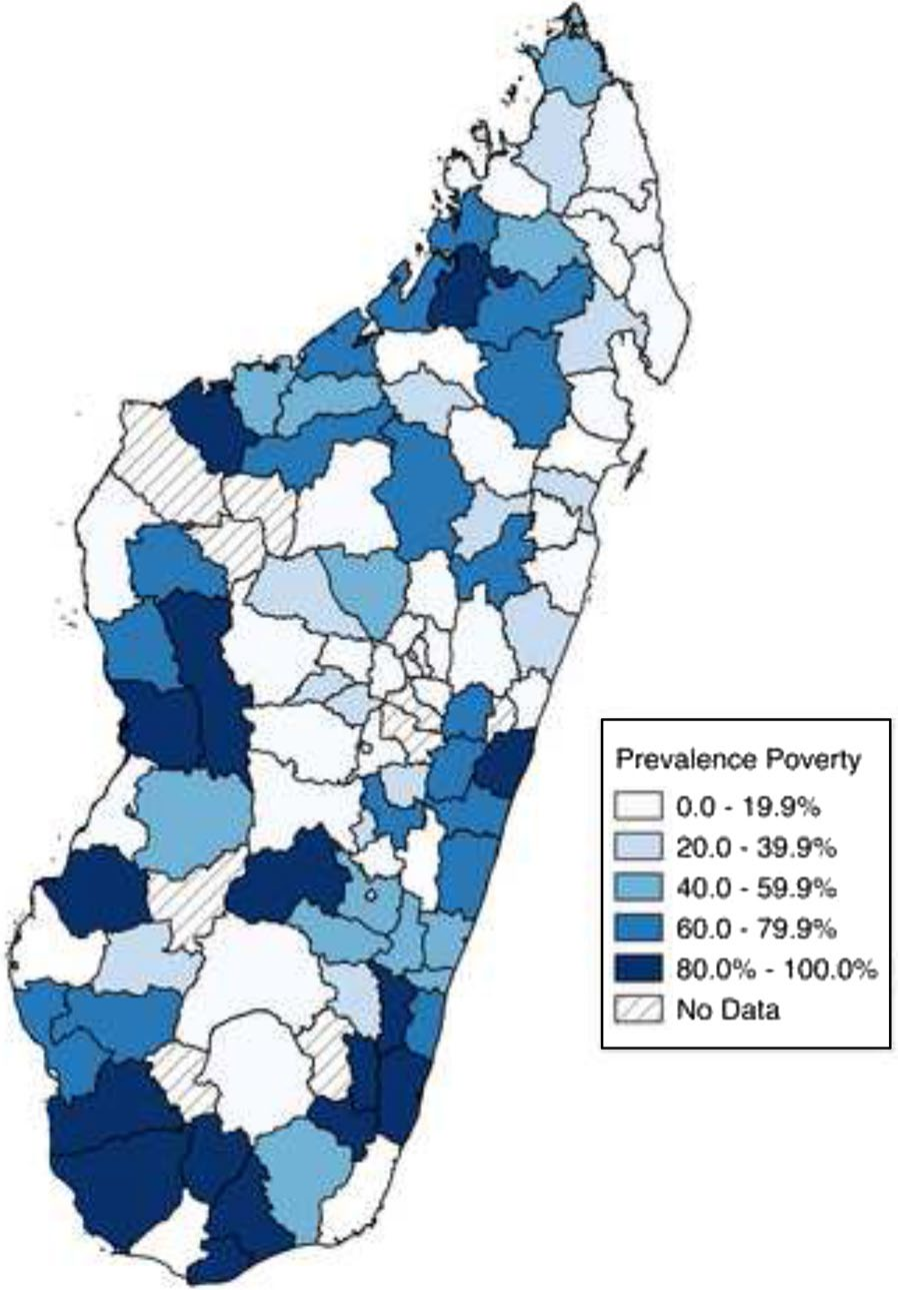
Prevalence of poverty (% household wealth in poorer to poorest categories) at the district level. Madagascar Malaria Indicators Survey 2011, 2013

Ecological comparisons show that the distribution of Malaria (Fig. 1) and socio-economic status (Fig. 2) were weakly associated (r = 0.24). Malaria risk is concentrated along the coastal regions, with the south, west, and eastern coasts facing the largest risks. Lower SES is concentrated in the southern part of the country, though there is also a cluster of poorer households in the north.

Odds ratios reveal associations between knowledge about malaria and both household wealth and mother’s education (Table 2). Specifically, mother’s education predicted all four domains of malaria knowledge, while household wealth predicted only whether women reported that mosquitoes transmitted malaria or that children were vulnerable.

**Table 2:** Odds ratios derived from multilevel logistic models estimating the influence of mother’s education and household wealth on knowledge about malaria, Madagascar Malaria Indicator Survey 2011, 2013

Fixed effects	Mosquitoes transmit malaria		Fever is a primary symptom		Children are vulnerable		Bed nets prevent malaria	
	OR	95 % CI	OR	95 % CI	OR	95 % CI	OR	95 % CI
Education								
None	1.00		1.00		1.00		1.00	
Primary	1.27	1.11, 1.46	1.16	1.01, 1.34	1.34	1.17, 1.53	1.18	1.02, 1.36
Secondary and higher	1.94	1.62, 2.31	1.53	1.29, 1.82	1.92	1.64, 2.25	1.76	1.48, 2.09
Wealth								
Poorest	1.00		1.00		1.00		1.00	
Poorer	1.08	0.92, 1.27	1.04	0.88, 1.23	0.95	0.81, 1.11	1.06	0.89, 1.26
Middle	1.42	1.18, 1.70	1.08	0.90, 1.30	1.26	1.06, 1.50	1.09	0.90, 1.32
Richer	1.45	1.19, 1.76	1.09	0.90, 1.33	1.33	1.11, 1.60	1.22	1.00, 1.50
Richest	1.88	1.48, 2.37	1.24	0.98, 1.55	1.55	1.25, 1.91	1.57	1.23, 1.99
Random effects								
SD (district)	0.16	0.08, 0.30	0.15	0.08, 0.27	0.15	0.08, 0.27	0.29	0.18, 0.47
SD (cluster)	0.25	0.18, 0.36	0.24	0.17, 0.34	0.25	0.18, 0.34	0.43	0.33, 0.56
Pseudo-R^2^	0.052	<0.001	0.026	<0.001	0.046	<0.001	0.135	<0.001
N	8,279		8,279		8,279		8,279	

All models adjust for mother’s age, year and month of interview, rural areas, household status, and whether women are currently pregnant

*OR* odds ratio, 95 % *CI* 95 % confidence interval, *SD*(*x*) standard deviation of x

Next, odds ratios examining efforts to prevent or treat malaria (Table 3) suggest that education and wealth were associated with mothers’ use of antimalarial prevention during pregnancy. However, while being in the highest SES category was strongly associated with seeking treatment for fevers, mother’s education was not associated with whether children slept under a treated bed net the previous night or whether or not treatment was sought for childhood fevers. Models fit the data examining use of treated bed nets well, with significant predictors being month, year, age of child, and both district- and community-level random intercepts.

**Table 3:** Odds ratios derived from multilevel logistic models providing associations between education and wealth on efforts to prevent and treat malaria, Madagascar Malaria Indicator Survey 2011, 2013

Fixed effects	IPTp used during pregnancy		Child slept under a treated net previous night		Child taken for treatment during last fever	
	OR	95 % CI	OR	95 % CI	OR	95 % CI
Education						
None	1.00		1.00		1.00	
Primary	1.62	1.17, 2.23	0.95	0.52, 1.72	0.74	0.33, 1.64
Secondary and higher	2.58	1.61, 4.12	0.87	0.41, 1.83	1.81	0.63, 5.18
Wealth						
Poorest	1.00		1.00		1.00	
Poorer	1.62	1.13, 2.33	1.05	0.52, 2.12	1.90	0.72, 4.96
Middle	2.97	1.79, 4.91	1.03	0.47, 2.27	1.02	0.36, 2.93
Richer	3.14	1.82, 5.42	0.71	0.30, 1.70	1.72	0.56, 5.26
Richest	3.62	1.90, 6.91	0.33	0.12, 0.95	6.43	1.53, 27.09
Random effects						
SD (district)	1.33	0.61, 2.89	24.09	15.65, 37.10	0.67	0.16, 2.79
SD (cluster)	1.01	0.49, 2.08	10.42	7.59, 14.31	1.24	0.26, 5.91
SD (household)	4.10	1.41, 11.9	23.32	18.48, 29.44	10.25	5.06, 20.78
Pseudo-R^2^	0.080		0.400		0.045	
N	4,682		7,850		1,055	

All models adjust for mother’s age, year and month of interview, rural areas, household status, and whether women are currently pregnant

*OR* odds ratio, 95 % *CI*: 95 % confidence interval, *SD* (*x*) standard deviation of x

Odds ratios provided in Table 4 show that education and wealth were associated with prevalence of malaria. Having a mother with a secondary education decreased the chances of malaria by more than half, while living in the richest households also led to a decrease in the likelihood of malaria. Models predicted 32.3 % of the variance in malaria prevalence. In these data, other covariates, including age of child, sex of child, and rural residence, along with random intercepts at district, community, and household levels. Covariates were significantly associated with prevalence of malaria.

**Table 4:** Odds ratios derived from multilevel logistic models estimating association between education and wealth on prevalence of malaria, Madagascar Malaria Indicator Survey 2011, 2013

Fixed effects	Prevalence	
	OR	95 % CI
Education		
None	1.00	
Primary	0.83	0.58, 1.19
Secondary and higher	0.51	0.28, 0.95
Wealth		
Poorest	1.00	
Poorer	0.71	0.47, 1.06
Middle	0.61	0.39, 0.97
Richer	0.25	0.13, 0.49
Richest	0.25	0.10, 0.64
Random effects		
SD (district)	1.55	0.82, 2.93
SD (cluster)	1.58	0.93, 2.70
SD (household)	1.18	0.54, 2.60
Pseudo-R^2^	0.323	
AIC	2,046	
N	6,879	

All models adjust for mother’s age, year and month of interview, rural areas, household status, and whether women are currently pregnant

*OR* odds ratio, 95 % *CI* 95 % confidence interval, *SD*(*x*) standard deviation of x

### Sensitivity analyses

Odds ratios derived from sample-weighted logistic regression (Additional file 1) supported these results, however, notable differences were evident. First, MLLM models provided fit better than did those not adjusting for community- and regional-level clustering: Pseudo-R^2^ derived from MLLM average a 252 % (ranging 169–426 %) improvement; likelihood ratio tests suggested that MLLM significantly improves on logistic regression in all cases (P < 0.001). Substantive results differed somewhat in that logistic regression analyses underestimated the influence of socio-economic factors, and estimated wider confidence intervals. Crucially, while logistic estimates of the influence of socio-economic factors on bed net usage showed a protective effect of higher wealth and knowledge on bed net usage, this influence largely disappeared when accounting for regional variation. Incorporating covariates including, household size, age and sex of household head, did little to influence the results shown here. Heckman corrections did not change estimates shown here. Finally, sensitivity analyses using rapid diagnostic testing rather than microscopic testing to identify malarial cases showed similar results.

## Discussion

Sociological theory suggests that when effective prevention or treatment exists for a disease then social actors use whatever resources they have, including money, knowledge, power, prestige, and beneficial social connections to access those technologies and mitigate their risk of disease [4, 5, 34–36]. Prior social epidemiological research has applied this theory to examine the role of SES in preventive medicine uptake in the USA [37], and Madagascar [20]. This study took a broad view by examining the role that socio-economic factors play in predicting the risk of risks. Results suggest that household socio-economic status and mother’s education play multiple potential roles, from geographic clustering of households to the prediction of knowledge about preventive efforts to curtail and the prevalence of malaria.

### Poverty versus social standing

Malaria has been called a ‘disease of poverty’ [15, 38]. This term, while generally used to highlight diseases that are susceptible to social inequalities, carries the implication that this unequal disease burden “finds its roots in the consequences of poverty, such as poor nutrition, indoor air pollution and lack of access to proper sanitation and health education” (pg 4) [38]. Marmot [39] famously distinguished between diseases of poverty and diseases of social standing by highlighting the dose–response association between malaria knowledge, treatment and prevention that is distributed across a population, and not solely risk factors among the poorest individuals. Indeed, many diseases in developed countries are subject to gradients in social inequality that have been attributed to social inequalities in access to and knowledge about prevention.

### Methodological considerations

Prior work examining the multilevel nature of social inequalities in disease have noted that socio-economic factors determine both where people live and the distribution of risk [18, 19]. In these analyses, cluster- and district-level variability helped to explain more variation in the outcomes than did SES and covariates alone, and also supported the view that socio-economic factors influence malaria. Yet, incorporating random effects estimates led to substantial improvements in model fit while also modifying some of the associations between wealth, education and malarial risk factors. Such results suggest that social inequalities arise in part through geographic clustering of socio-economic status and malarial risk.

### Policy efforts and recommendations

Starting in 2008, there has been a substantial and sustained effort both at the national and international levels to freely distribute long lasting insecticide-treated bed nets to all households throughout Madagascar [40]. These efforts have generally been fruitful with regard to bed nets in Madagascar, where one distribution campaign found that 99.5 % of households used bed nets one month following distribution campaigns [41]. Such distribution campaigns have previously been shown to influence bed net usage irrespective of socio-economic status [42]. While it is likely that universal distribution of bed nets made a substantial difference to the health of the population, it also has the potential to reduce inequalities. Indeed, analyses have shown that in 2007–2008, prior to such campaigns, ownership of bed nets in areas was often sparse and strongly associated with socio-economic inequality [25].

Policy efforts often focus on whether to use universal or targeted approaches to improve public health [43]. Critiques suggest that one problem with such focuses is that they ignore how socio-economic inequalities may influence results [44]. Yet, a randomized control trial found that prior inequalities in wealth caused differential uptake in new health information [45]. Inequalities in knowledge about malaria can reduce the effectiveness of many interventions because people do not know why or when to access and use them. For instance, in these data 71.7 % thought that fever was a primary symptom for malaria, while only 59.2 % believed that bed nets could be used to prevent malaria. In such cases, free prevention and treatment cannot be universally effective because a substantial proportion of the population does not know how to recognize or prevent disease. Simulation results suggest that universal policies often result in substantial improvements in population health but do little to improve socio-economic inequalities therein [46]. In the current study, bed nets were used by 69.4 % of children, despite the common misconception (among 40.8 % of women in this sample) that they do not prevent malaria. However, this usage was not subject to social inequalities to the same extent that other indicators were. This lack of inequality may be a result of such efforts to distribute nets and encourage their usage, and if so provide a type of intervention that may be both efficient and equitable.

Of the mechanisms found here, large inequalities were found stemming from differences in knowledge about malaria. Interventions often rely on school-based informational campaigns to improve knowledge [47]; however, a fifth of the women in these data report not having finished primary schooling. Such lack of schooling may result in greater inequalities if those with the least schooling are more likely to be excluded from such interventions. Socio-economic development has recently been suggested as a potential mechanism for both the reduction of inequalities and the control of malaria [14]. Further, while distribution and use of bed nets provide substantial preventative benefits in Madagascar across all SES groups, further efforts will be necessary to improve malaria control. These may need to come from sustained efforts to invest in human development alongside medical interventions.

## Limitations

This study is limited by a lack of consideration for causal direction. In particular, there was an association between regional inequalities in malaria risk and continued poverty, suggesting the potential for reverse causation [13]. Malaria may, over the long term, reduce productivity among individuals, for instance by increasing expenditure on care, and reducing human capital through increased absenteeism and lowered earnings in malaria-endemic areas. Malaria is also known to result in substantial comorbidities including, for example, anaemia, and neurological problems [48]. Malaria is endemic within 96 % of regions in Madagascar [40], and MLLM is effective at reducing between-cluster inequalities in the geographic distribution of the risk of malaria and instead focuses attention on social inequalities within geographic regions where the likelihood of malarial infection is more uniformly distributed than it is nationwide. Indeed, such associations likely account for the differences between MLLM and logistic results. However, such recursive risks would drastically increase the influence of social inequality over time and make malaria both more difficult and more important to control.

Examining malaria in Madagascar, an island nation with a unique heritage and geography and a large rural population living in poverty, limited the generalizability of this study. Despite being poor in absolute terms, clear social inequalities indicate that results may not be unique to Madagascar but may reflect processes of relative inequality noted by social epidemiologists for diseases worldwide [49].

While models are robust to data that are missing at random, they may be biased if data are missing because children died due to incident malaria. Indeed, because knowledge about malaria etiology, as well as access to prevention and treatment for malaria, are socio-economically graded, it is likely that those children whose data are missing are more likely to be among mothers with lower education living in poorer households within areas of lower socio-economic status. These analyses tried to account for this censorship through use of Heckman corrections, and found similar results. Nevertheless, such patterns of missing data suggest that these results may be conservative.

While these limitations are substantial, this study has a number of strengths that make it unique. These data are some of the most recent and up-to-date, suggesting that they are representative of the current situation on the ground in Madagascar. Sample sizes are maximized by the pooling of 2 years of data from the MIS in Madagascar, drastically improving power to examine the influence of socio-economic status. Moreover, these methods represent substantial improvements over logistic regression methods, both because they provide models that better fit these data and because they account for shared but unobserved factors that influence the risk of malaria and the risk of risk factors for malaria.

## Conclusion

In Madagascar, 5.8 % of children die before their fifth birthday and malaria is the underlying cause for 7 % (2010 est.) of these deaths [23]. However, preventing malaria and reducing the burden of disease requires a multi-pronged action plan. The plan currently focuses on distributing bed nets, free diagnostic testing among all patients, free or low-cost treatment, routine surveillance, and access to effective antimalarial drugs [50]. The effectiveness of intervention requires that individuals access and ultimately choose to use preventive technologies to reduce malarial transmission. Substantial variation in the indicators of disease were found: for example 29 % of mothers did not recognize fever as a main symptom for malaria while nearly 50 % did not think that children were vulnerable to the disease. Such results may not be particularly surprising in Madagascar, where nearly 21 % of women aged 15–49 lacked primary schooling. Nevertheless, action to prevent or treat malaria requires a broad range of interventions, from improving knowledge about how to effectively avoid malaria to improving access to bed nets and other prevention or treatment that may improve outcomes when knowledge is lacking.

## Additional file

10.1186/s12936-015-1010-y Odds ratios linking malarial knowledge and use of preventive medicine derived from logistic regression, Malaria Indicator Survey 2011–2013.
