# Supplementary material for: Social inequalities in malaria knowledge, prevention and prevalence among children under 5 years old and women aged 15–49 in Madagascar
Source: Malar J. 2015 Dec 12;14:499. doi: 10.1186/s12936-015-1010-y (PMC4676822; doi:10.1186/s12936-015-1010-y)
Supplement: Supplementary file 1 — 10.1186/s12936-015-1010-y Odds ratios linking malarial knowledge and use of preventive medicine derived from logistic regression, Malaria Indicator Survey 2011–2013. [file 12936_2015_1010_MOESM1_ESM.docx]

|  |  | **Mosquitos transmit malaria** | |  | **Fever is a primary symptom** | |  | **Children are vulnerable** | |  | **Bed nets prevent malaria** | |  | **IPTp used during pregnancy** | |  | **Child slept under a bed net prior night** | |  | **Child taken for treatment for last fever** | |  | **Prevalence of malaria** | |
| --- | --- | --- | --- | --- | --- | --- | --- | --- | --- | --- | --- | --- | --- | --- | --- | --- | --- | --- | --- | --- | --- | --- | --- | --- |
|  |  | **OR** | **95% C.I.** |  | **OR** | **95% C.I.** |  | **OR** | **95% C.I.** |  | **OR** | **95% C.I.** |  | **OR** | **95% C.I.** |  | **OR** | **95% C.I.** |  | **OR** | **95% C.I.** |  | **OR** | **95% C.I.** |
| Education | None | 1.00 |  |  | 1.00 |  |  | 1.00 |  |  | 1.00 |  |  | 1.00 |  |  | 1.00 |  |  | 1.00 |  |  | 1.00 |  |
|  | Primary | 1.11 | 0.92, 1.34 |  | 1.29 | 1.08, 1.54 |  | 1.37 | 1.14, 1.64 |  | 1.15 | 0.94, 1.39 |  | 1.23 | 1.01, 1.50 |  | 0.66 | 0.81, 0.53 |  | 1.26 | 0.82, 1.93 |  | 1.07 | 0.78, 1.46 |
|  | Secondary | 1.89 | 1.51, 2.37 |  | 1.60 | 1.27, 2.01 |  | 1.73 | 1.39, 2.15 |  | 1.68 | 1.35, 2.10 |  | 1.74 | 1.34, 2.25 |  | 0.70 | 0.91, 0.53 |  | 1.84 | 1.06, 3.20 |  | 2.29 | 1.32, 3.95 |
| Wealth | Poorest | 1.00 |  |  | 1.00 |  |  | 1.00 |  |  | 1.00 |  |  | 1.00 |  |  | 1.00 |  |  | 1.00 |  |  | 1.00 |  |
|  | Poorer | 1.12 | 0.93, 1.36 |  | 1.01 | 0.83, 1.24 |  | 0.92 | 0.76, 1.12 |  | 1.12 | 0.87, 1.44 |  | 1.27 | 0.99, 1.63 |  | 1.00 | 1.31, 0.77 |  | 1.29 | 0.73, 2.31 |  | 1.16 | 0.83, 1.63 |
|  | Middle | 1.36 | 1.10, 1.68 |  | 1.06 | 0.83, 1.34 |  | 1.30 | 1.06, 1.61 |  | 1.08 | 0.82, 1.42 |  | 1.57 | 1.24, 1.99 |  | 1.00 | 1.05, 0.62 |  | 1.09 | 0.61, 1.94 |  | 1.43 | 0.99, 2.06 |
|  | Richer | 1.58 | 1.26, 1.99 |  | 1.22 | 0.95, 1.55 |  | 1.38 | 1.11, 1.72 |  | 1.15 | 0.88, 1.50 |  | 1.51 | 1.17, 1.94 |  | 0.81 | 0.96, 0.55 |  | 1.17 | 0.65, 2.08 |  | 4.16 | 2.41, 7.19 |
|  | Richest | 1.78 | 1.37, 2.31 |  | 1.42 | 1.06, 1.90 |  | 1.60 | 1.23, 2.08 |  | 1.11 | 0.81, 1.53 |  | 1.24 | 0.93, 1.66 |  | 0.72 | 0.83, 0.44 |  | 1.54 | 0.82, 2.90 |  | 5.15 | 2.13, 12.42 |
|  |  |  |  |  |  |  |  |  |  |  |  |  |  |  |  |  |  |  |  |  |  |  |  |  |
| Pseudo-R^2^ |  | 0.031 |  |  | 0.010 |  |  | 0.020 |  |  | 0.076 |  |  | 0.036 |  |  | 0.111 |  |  | 0.027 |  |  | 0.076 |  |
| N |  | 8279 |  |  | 8279 |  |  | 8279 |  |  | 8279 |  |  | 4682 |  |  | 7850 |  |  | 1055 |  |  | 7644 |  |
